# Sodium Butyrate Protects N2a Cells against A*β* Toxicity In Vitro

**DOI:** 10.1155/2020/7605160

**Published:** 2020-04-15

**Authors:** Jingxuan Sun, Boyu Yuan, Yancheng Wu, Yuhong Gong, Wenjin Guo, Shoupeng Fu, Yongxin Luan, Wei Wang

**Affiliations:** ^1^Key Laboratory of Zoonosis Research, Ministry of Education, College of Veterinary Medicine, Jilin University, Changchun 130062, China; ^2^Department of Pharmacology, College of Basic Medical Science, Jilin University, Changchun 130012, China; ^3^First Clinical Hospital of Jilin University, Changchun, China; ^4^Innovative Institute of Animal Healthy Breeding, Key Laboratory of Waterfowl Healthy Breeding of Guangdong Province, College of Animal Sciences and Technology, Zhongkai University of Agriculture and Engineering, Guangzhou, China

## Abstract

Alzheimer's disease (AD) is a common neurodegenerative disease. A*β* plays an important role in the pathogenesis of AD. Sodium butyrate (NaB) is a short-chain fatty acid salt that exerts neuroprotective effects such as anti-inflammatory, antioxidant, antiapoptotic, and cognitive improvement in central nervous system diseases. The aim of this study is to research the protective effects of NaB on neurons against A*β* toxicity and to uncover the underlying mechanisms. The results showed that 2 mM NaB had a significant improvement effect on A*β*-induced N2a cell injury, by increasing cell viability and reducing ROS to reduce injury. In addition, by acting on the GPR109A receptor, NaB regulates the expression of AD-related genes such as APP, NEP, and BDNF. Therefore, NaB protects N2a cells from A*β*-induced cell damage through activating GPR109A, which provides an innovative idea for the treatment of AD.

## 1. Introduction

Alzheimer's disease (AD) belongs to neurodegenerative diseases, which is characterized by extracellular amyloid deposition and intracellular neurofibrillary tangles [1–3]. Clinically, it shows through generalized dementia such as memory impairment, aphasia, disuse, loss of recognition, impairment of visuospatial skills, executive dysfunction, and personality or behavioral changes. Although there is still controversy about the pathogenesis of AD, the more accepted hypothesis is that the abnormal decomposition or production of the amyloid precursor protein (APP) leads to the deposition of neurotoxic *β*-amyloid (A*β*) oligomers, triggering an amyloid cascade reaction which finally leads to neurodegeneration [4–7]. Undisputed evidence says there are insoluble amyloid plaques formed by A*β* deposition in the brain of AD patients [8]. Although significant progress has been made in the study of its pathogenesis, the current clinical treatment methods can only delay the development of AD and cannot reverse the existing neuron damage. Therefore, it is the primary task to find effective therapeutic targets and therapeutic drugs effectively, to prevent damage to the nervous system, and to repair damaged nerve cells under the toxicity of A*β* plaques, thereby improving cognitive ability in early AD patients.

Butyrate is under a wide range of biological functions. Studies have shown that butyrate plays an active role in brain disorders in a variety of neurodegenerative diseases such as Alzheimer's disease, Parkinson's disease, and Huntington's disease [9–12]. Sodium butyrate (NaB) is a common form of butyrate. A study by Sun et al. showed that NaB protects brain against amphetamine-induced oxidative stress in rats [13]. Some studies indicate that the A*β*-mediated oxidative stress is a major factor in the pathology of AD [14, 15]. In an AD mouse model, NaB significantly improves the associative memory of APPPS1-21 mice (Alzheimer's mice) [16].

G protein-coupled receptors are widely found in mammals and are encoded by approximately 800 genes. Typical natural ligands for G protein-coupled receptors include hormones, mediators, neurotransmitters, polypeptides, amino acids, and ions. Studies have demonstrated that in the process of energy metabolism, certain nutrients or their metabolites, which are the basic raw materials, activate G protein-coupled receptors to regulate metabolism [17]. The GPR109A receptor, otherwise known as niacin receptor 1, belongs to the G protein-coupled receptor family. Niacin is the major ligand for GPR109A, but the physiological concentration of niacin does not reach the threshold required to activate the receptor [18], while butyrate, as a suitable candidate ligand, has the potential in binding the GPR109A receptor at a low concentration [19]. As a Gi/G0-coupled receptor, pertussis toxin (PTX) blocks the effects of GPR109A.

Therefore, this study was aimed at investigating the protective effect of NaB on N2a cells induced by A*β* in vitro and at exploring the mechanisms on how GPR109A is involved.

## 2. Materials and Methods

### 2.1. Cell Culture

Mouse neuroblastoma N2a cells were donated by the pathology laboratory of the College of Veterinary Medicine, Jilin University. All cells were cultured in DMEM medium (Gibco, Grand Island, NY 14072, USA) containing 10% fetal bovine serum (FBS) (Clark Bioscience, USA) at 37°C in a humidified incubator with 5% CO_2_. N2a cells were cultured in a 60 mm × 15 mm cell culture dish (Life Science, Oneonta, USA).

### 2.2. Treatment of N2a Cells

A*β*_25-35_ (synthesized by Shanghai Sangon Biological Engineering Technology & Services Co.) was diluted in distilled water at a concentration of 16 mmol/L and was maintained at 37°C for 7 days to preage the peptide. N2a cells were divided into four groups: the control (NT) group, the NaB (2 mM) group, the A*β*_25-35_ (40 *μ*mol/L) treatment group, and the A*β*_25-35_ (40 *μ*mol/L) with NaB (2 mM) pretreatment group. Cells were starved for 4 h and then pretreated with 2 mM NaB for 2 h before A*β*_25-35_ was added. RNA or protein from N2a cells was extracted after 24 h. Each experiment was repeated three times.

### 2.3. Cell Counting Kit-8 Assay

N2a cells were centrifuged and seeded in 96-well plates at a density of 2 × 10^4^ cells/mL. 200 *μ*L of PBS was added, and the cells were incubated at 37°C. After 24 hours, the complete medium was removed, and 200 *μ*L prewarmed DMEM basal medium was added for cell starvation. Subsequently, NaB was added after 4 h to pretreat the cells, and after 2 h, A*β*_25-35_ was added for stimulation. There were 5 replicates for each test group. After 24 h of treatment, 10 *μ*L CCK8 (Saint-Bio, Shanghai, China) was added to each well. After 1 h, absorbance (OD) was measured at 450 nm in a microplate reader.

### 2.4. Reactive Oxygen Species Assay Kit

N2a cells were seeded in a 96-well plate at a liquid volume of 2 × 10^4^ cells/mL, and each treatment group was set to five repeats. After culturing for 24 h, replace with DMEM incomplete medium to starve the cells for 4 h. Then, 2 mM NaB was added for preprotection for 2 h. And the cells were stimulated with A*β*_25-35_ at a dose of 40 *μ*M/well for 24 h. Thereafter, DCFH-DA was diluted with noncomplete medium at a ratio of 1 : 2000 and cultured for 40 min. The detection wavelength is 488 nm excitation wavelength and 525 nm emission wavelength.

### 2.5. Real-Time qPCR

RNA extraction, reverse transcription, and RT-PCR were performed following our previously published procedure [20]. The primer sequences are shown in Table 1.

### 2.6. Western Blot

Total proteins were extracted from N2a cells following the procedure we described previously [20]. Subsequently, western blotting was performed according to the standard protocol [20]. Antibody information is as follows: specific antibodies including tubulin (1 : 4000), APP (1 : 5000; A8717, Sigma-Aldrich), NEP (1 : 1500; 18008-1-AP, Proteintech), BDNF (1 : 1000; 28205-1-AP, Proteintech), GPR109A (1 : 500; NBP1-92180,NOVUS), goat anti-rabbit IgG-HRP (1 : 3000; Santa Cruz Biotechnology), and goat anti-mouse IgG-HRP antibody (1 : 3000; Santa Cruz Biotechnology).

### 2.7. Statistical Analysis

The results are expressed as the mean ± SD (or mean ± SEM). Statistical analysis was performed using Student's *t*-test or one-way ANOVA followed by Tukey's post hoc test using Prism5.0 (GraphPad Software). *P* values < 0.05 were considered as statistically significant.

## 3. Result

### 3.1. NaB Regulates a Variety of AD-Related Genes in N2a Cells

**Figure 1:**
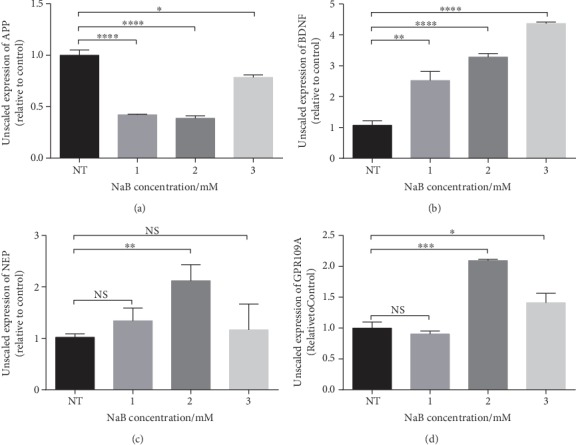
NaB regulates a variety of AD-related genes in N2a cells. N2a cells were treated with 0, 1, 2, and 3 mM NaB for 24 hours. (a–d) The mRNA expressions of APP, BENF, NEP, and GPR109A were assessed by RT-PCR (*n* = 3, means ± SD, Student's *t*-test, ^∗^*P* < 0.05, ^∗∗^*P* < 0.01, ^∗∗∗^*P* < 0.001, and ^∗∗∗∗^*P* < 0.0001). The effect of NaB on N2a cells is most obvious when the concentration is 2 mM.

According to our previous results, we found that NaB has effects on multiple cells in regulating gene expression. In order to examine the effect of NaB on N2a cells and obtain the optimal concentration, we detected AD-related genes in N2a cells by RT-PCR, under the treatment of 1, 2, and 3 mM NaB. Compared with the control group, 2 mM NaB had the most significant inhibitory effect on APP (Figure 1(a)) and the promotion effect on NEP and BDNF (Figures 1(b) and 1(c)). In the subsequent experiments, we chose 2 mM as the appropriate concentration of NaB. In addition, we found that NaB also significantly increases the expression of GPR109A (Figure 1(d)).

### 3.2. NaB Shows Protective Effect on A*β*-Induced N2a Cell Viability Decrease

**Figure 2:**
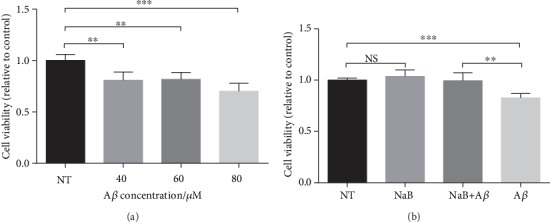
NaB shows protective effect on A*β*-induced N2a cell viability decrease. (a) Effect of A*β*_25-35_ on N2a cell viability. Cells were incubated with 0, 40, 60, or 80 *μ*M A*β*_25-35_ for 24 hours (*n* = 4, means ± SD, Student's *t*-test, ^∗∗^*P* < 0.01, and ^∗∗∗^*P* < 0.001). (b) Effect of 2 mM NaB pretreatment on cell viability challenged by 40 *μ*M A*β*_25-35_ for 24 hours. Cells were starved for 4 h and then pretreated with 2 mM NaB for 2 h before A*β*_25-35_ was added. Cell viability was analyzed by the CCK-8 assay (*n* = 4, means ± SD, Student's *t*-test, ^∗∗^*P* < 0.01, and ^∗∗∗^*P* < 0.001). When the concentration of A*β* is 40 *μ*M, it had caused obvious damage to the N2a cells. After pretreatment with 2 mM NaB, cell viability was restored.

A*β* oligomer is cytotoxic and can significantly reduce cell vitality. When the concentration of A*β* reached 40 *μ*M, A*β* caused a significant decrease in cell viability (Figure 2(a)). To investigate the protective effect of NaB on N2a cells, we used CCK-8 to detect cell viability under A*β* oligomer incubation. The results show that compared with the control group, A*β* dramatically decreased cell viability, while NaB had no significant effect on cell viability (Figure 2(b)). Compared with A*β* treatment, NaB protected N2a cells on cell viability significantly under A*β* oligomer incubation (Figure 2(b)).

### 3.3. NaB Attenuates A*β* Toxicity and Maintains Mitochondrial Respiratory Function in N2a Cells

**Figure 3:**
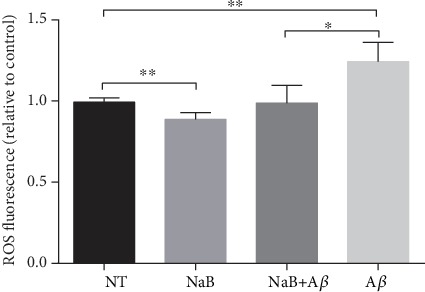
NaB attenuates A*β* toxicity and maintains mitochondrial respiratory function in N2a cells. Effect of 2 mM NaB pretreatment on ROS challenged by 40 *μ*M A*β*_25-35_ for 24 hours. Cells were starved for 4 h and then pretreated with 2 mM NaB for 2 h before A*β*_25-35_ was added. The ROS was assessed by a ROS assay kit (*n* = 4, means ± SD, Student's *t*-test, ^∗^*P* < 0.05, and ^∗∗^*P* < 0.01). When the concentration of A*β* is 40 *μ*M, it had led to increased ROS in N2a cells. After pretreatment with 2 mM NaB, ROS was reduced to normal levels.

ROS are mainly produced in the mitochondrial electron-transport chain. The accumulation of ROS reflects the damage degree of mitochondrial function. Using a ROS assay kit, we found that A*β* made N2a cells produce a large amount of ROS (Figure 3), while treating with NaB significantly inhibited the production of A*β*-induced ROS in N2a cells (Figure 3).

### 3.4. NaB Suppresses APP Expression and Increases BDNF/NEP Level in GPR109A-Dependent Manner

**Figure 4:**
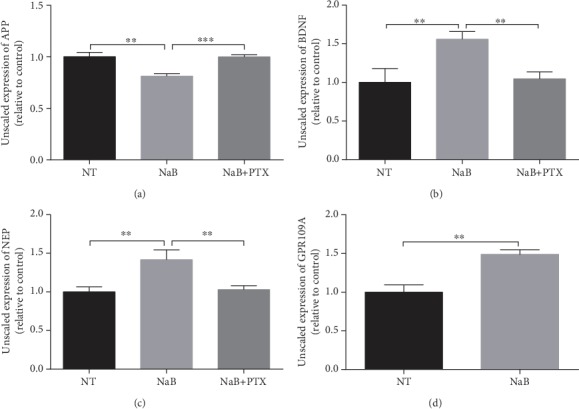
NaB suppresses APP expression and increases the BDNF/NEP level in a GPR109A-dependent manner in the mRNA level. The mRNA expressions of APP, BENF, NEP, and GPR109A were assessed by RT-PCR. The N2a cells were pretreated for 1 hour with 1 mM PTX and then incubated for 24 hours with 2 mM NaB. (a–d) PTX inhibited NaB-mediated change in APP, BDNF, NEP, and GPR109A mRNA expression (*n* = 3, means ± SD, Student's *t*-test, ^∗^*P* < 0.05, ^∗∗^*P* < 0.01, and ^∗∗∗^*P* < 0.001). 2 mM NaB reduced transcription of APP and increased transcription of BDNF and NEP. At the same time, NaB promoted the transcription of the GPR109A.

**Figure 5:**
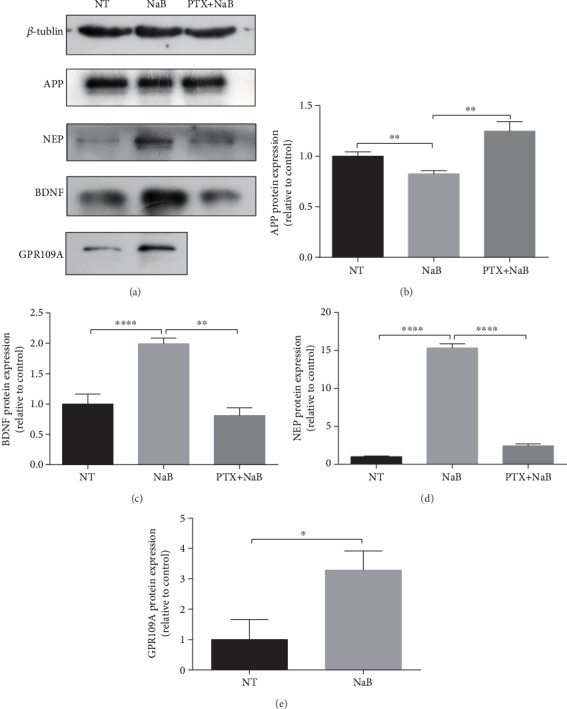
NaB suppresses APP expression and increases the BDNF/NEP level in a GPR109A-dependent manner in the protein level. The N2a cells were pretreated for 1 hour with 1 mM PTX and then incubated for 24 hours with 2 mM NaB. (a) Protein expression of APP, NEP, BDNF, and GPR109A in N2a cells was assessed by western blotting (*n* = 3); *β*-tubulin was used as an internal loading control. (b–e) Quantitative analysis of the expression of APP, BDNF, NEP, and GPR109A of no treatment (NT), NaB-treated (NaB), and PTX and NaB-treated (PTX+NaB) (*n* = 3, means ± SD, Student's *t*-test, ^∗^*P* < 0.05, ^∗∗^*P* < 0.01, ^∗∗∗^*P* < 0.001, and ^∗∗∗∗^*P* < 0.0001). 2 mM NaB reduced expression of APP and increased expression of BDNF and NEP. Simultaneously, NaB promoted the expression of the GPR109A.

We have proven that NaB has a positive effect on A*β*-induced neurocyte injury. To investigate whether this effect is mediated by GPR109A, we treated N2a cells with PTX, which inhibits the G protein and its physiological function in the signaling pathway. We found both in the mRNA level (Figure 4) and in the protein level (Figure 5), compared with the NaB group; after PTX treatment, the inhibitory effect of NaB on APP and the promoting effect on NEP and BDNF were alleviated. Meanwhile, NaB significantly increased GPR109A gene expression. These results indicate that the GPR109A is necessary for mediating AD-related gene expression regulated by NaB.

## 4. Discussion

Butyrate reportedly exerts numerous beneficial effects on the gut, immune system, central nervous system, and cardiovascular system [21]. Previous studies have shown that butyrate has a positive effect on both astrocytes [22] and microglia [23–26] of the central nervous system. Moreover, previous research in our laboratory shows that *β*-hydroxybutyric acid as a short-chain fatty acid, the same with butyrate, protected dopaminergic neurons through inhibiting microglia-mediated neuroinflammation both in vitro and in vivo [27] and limited the excessive activation of microglia and inhibited the production of inflammatory cytokines in 5XFAD mice [28]. However, the effects and mechanism of butyrate on neuronal cell regulation, especially under AD conditions and A*β* toxicity, need to be studied well and clarified. Our results suggest that NaB reduces A*β*-induced N2a cell damage by reducing ROS. NaB inhibits the expression of APP, promotes the expression of NEP and BDNF in N2a cells by activating G proteins, especially GPR109A. These results suggest that NaB may be a candidate for the cure of AD.

A*β* is produced by the cleavage of APP by *β*- and *γ*-secretase [29]. Previous studies have found that the overexpression of APP led to the increase of A*β*, which is closely related to the occurrence of AD [30–43]. It has been shown that BHBA can improve cognitive behavior in an Alzheimer's disease mouse model. In the course of its action, the expression of APP decreased [28]. On the one hand, NEP can effectively disintegrate A*β* and slow down the development of amyloidosis [44–46]. Therefore, the dysregulation of NEP activity can promote the deposition of A*β* and affect the development of AD [47–49]. According to the research of Wei et al., it is shown that pratensein can promote the expression of NEP and reduce the deposition of A*β* [50]. In addition, early studies have shown that during the occurrence and development of AD, the expression of BDNF is reduced both in mRNA and protein levels [51–53]. Increased BDNF in the blood slowed AD development and cognitive decline [51, 53–55]. Studies have shown that the expression of BDNF is significantly reduced in an A*β*_1-42_-induced rat model, while pratensein can reverse the decrease of BDNF expression [50]. In our study, NaB can reduce the production of APP in mRNA and protein levels, thereby reducing the formation of A*β*. Simultaneously, NaB can also promote the generation of NEP, sequentially promoting the degradation of A*β*. In addition, NaB can also promote the production of BDNF and reduce A*β*-induced cell damage.

It has been proved that the deposition of A*β* in mitochondria and the promotion of ROS under the action of various metal ions are important features of AD during the development of AD [56–58]. The analysis of mouse models and autopsy of AD patients showed that mitochondrial dysfunction led to increased ROS, further exacerbating mitochondrial dysfunction, which further resulted in the deposition of A*β*. In AD pathology, with the accumulation of ROS, A*β* deposition caused mitochondrial dysfunction, which can lead to neuronal cell death [59]. Our study shows that NaB can reduce the production of ROS, thereby maintaining mitochondrial function and reducing A*β*-induced cell damage.

We showed the strong evidence that NaB inhibits the expression of APP, promotes the expression of NEP and BDNF, reduces the accumulation of ROS, and reverses the decrease of cell activity caused by A*β*, by activating G protein, especially GPR109A, which may provide new ideas for the treatment of AD.

## Figures and Tables

**Table 1:** Primers for real-time PCR.

Gene	Sequences	Length (bp)
GPR109A	Forward: 5′-CCGTCGTGTAGTCTGTCTCGTG-3′	119
	Reverse: 5′-GCTGCGGTTATTGTTGGACT-3′	

APP	Forward: 5′-CCAAGGAGGGCATCTTGCAG-3′	138
	Reverse: 5′-TGTGGGTGTGTGTCTTGCAC-3′	

BDNF	Forward: 5′-CGACGACATCACTGGCTGAC-3′	124
	Reverse: 5′-AGCATCACCCGGGAAGTGTA-3′	

NEP	Forward: 5′-CTA CCG GCC AGA GTA TGC AG-3′	133
	Reverse: 5′-TTG CGG CAA TGA AAG GCA TC-3′	

GAPDH	Forward: 5′-GCCATCACTGCCACCCAGAA-3′	153
	Reverse: 5′-GCCAGTGAGCTTCCCGTTGA-3′	

## Data Availability

“The data used to support the findings of this study are available from the corresponding author upon request.”
